# The Importance of the Validation of M/EEG With Current Biomarkers in Alzheimer's Disease

**DOI:** 10.3389/fnhum.2019.00017

**Published:** 2019-02-05

**Authors:** Fernando Maestú, Pablo Cuesta, Omar Hasan, Alberto Fernandéz, Michael Funke, Paul E. Schulz

**Affiliations:** ^1^Laboratory of Cognitive and Computational Neuroscience, Center for Biomedical Technology, Universidad Complutense and Universidad Politécnica de Madrid, Madrid, Spain; ^2^Department of Experimental Psychology, Universidad Complutense de Madrid, Madrid, Spain; ^3^Magnetic Source Imaging Unit, Department of Pediatrics, McGovern Medical School, University of Texas Health Science Center, Houston, TX, United States; ^4^Electrical Engineering and Bioengineering Lab, Department of Industrial Engineering & IUNE Universidad de La Laguna, Tenerife, Spain; ^5^McGovern Medical School University of Texas Health Science Center, Houston, TX, United States; ^6^Department of Legal Medicine, Psychiatry, and Pathology, Universidad Complutense de Madrid, Madrid, Spain

**Keywords:** functional connectivity, mild cognitive impairment, EEG, MEG, healthy aging, Alzheimer's disease

## Abstract

Current biomarkers used in research and in clinical practice in Alzheimer's Disease (AD) are the analysis of cerebral spinal fluid (CSF) to detect levels of Aβ42 and phosphorylated-tau, amyloid and FDG-PET, and MRI volumetry. Some of these procedures are still invasive for patients or expensive. Electroencephalography (EEG) and Magnetoencephalography (MEG) are two non-invasive techniques able to detect the early synaptic dysfunction and track the course of the disease. However, in spite of its added value they are not part of the standard of care in clinical practice in dementia. In this paper we review what these neurophysiological techniques can add to the early diagnosis of AD, whether results in both modalities are related to each other or not, as well as the need of its validation against current biomarkers. We discuss their potential implications for the better understanding of the pathophysiological mechanisms of the disease as well as the need of performing simultaneous M/EEG recordings to better understand discrepancies between these two techniques. Finally, more studies are needed studying M/EEG with amyloid and Tau biomarkers.

## Introduction

Alzheimer's disease (AD) is the major cause of dementia in the elderly (Qiu et al., 2009). It is characterized by the accumulation of amyloid beta (Aβ) protein, the hyperphosphorylation of tau protein, and neuroinflammation, that are thought to lead to neuronal loss and synaptic dysfunction. Current biomarkers used in research and in clinical practice are the analysis of cerebral spinal fluid (CSF) to detect levels of Aβ42 and phosphorylated-tau, amyloid and FDG-PET, and MRI volumetry. Pathological changes associated with AD, such as amyloid deposition, start decades before the first clinical symptoms appear, and clinically-relevant functional loss may be irrecoverable once the disease process has progressed. As such, it is imperative to detect neuropathological changes, especially those at the synaptic level, as early in the development of the disease process as possible. While the increased amyloid burden can be detected in preclinical stages in subjects at risk of dementia (Dubois et al., 2016; Jack et al., 2017), hypometabolism, and cortical atrophy is typically not detected until the early dementia stages, such as prodromal dementia (Jack et al., 2013, 2017). Amyloid load has not been clearly associated with cognitive impairment, however, increased tau at CSF showed high correlation with cognitive deficit (Nelson et al., 2012; Vos et al., 2013). Amyloid PET and CSF are too invasive and expensive to use to screen large numbers of presymptomatic patients. Thus, a test is needed that can be used to screen large populations, demonstrated the onset of the neuropathology of the disease, that is easy to use and non-invasive, to be able to detect the initial stages of the disease and to track its course.

## Electrophysiological Techniques to Detect Presymptomatic AD

The analysis of human and animal brain tissues has shown that amyloid plaques may have a toxic effect on inhibitory terminals (Garcia-Marin et al., 2009) as well as for excitatory neurons (Pozueta et al., 2013; Wang et al., 2017). This phenomenon disrupts the normal balance between excitation and inhibition in neuronal activity, increasing neuronal excitability and damaging neural network function (Busche and Konnerth, 2016; Teipel et al., 2016; Sepulcre et al., 2017). Furthermore, hyperactivity of hippocampal neurons precedes amyloid plaque formation, suggesting that hyperactivity is one of the earliest dysfunctions in the pathophysiological cascade initiated by abnormal Aβ accumulation (Busche and Konnerth, 2016).

Various forms of p-Tau also alter the normal network functioning. Tau deposition disrupts the axonal microtubule architecture (Taniguchi et al., 2001) and its deposits correlates with cognitive impairment (Nelson et al., 2012; Vos et al., 2013; Albert et al., 2018) and network dysfunction (Schultz et al., 2017). This progressive loss of synapse efficiency and quantity, disrupts inter- and intra-regional communication, leading to the proposal that AD is a disconnection syndrome (Hardy and Selkoe, 2002; Delbeuck et al., 2003).

These pathophysiological processes have direct consequences on neural transmission and therefore can be detected by neurophysiological techniques. Synaptic dysfunction and disruption of connectivity, then, can be studied with Magnetoencephalography (MEG) and Electroencephalography (EEG) (M/EEG). MEG records the magnetic fields induced by intracellular post-synaptic activity (Hari and Puce, 2017) and the EEG is a direct measure of neuronal field potentials that can be used to assess the organization of brain functional architecture in AD (Stam, 2010). It is true that while their temporal resolution is high their ability to localize brain activity from deep sources is limited. However, the time-frequency resolution allows for detecting brain oscillatory activity which contains the “neural code” for the local and long-range transmission of information. MEG is reference free avoiding the need of a reference to close the electrical current needed in EEG which could modify the phase of the signals detected in each electrode. If synaptic dysfunction and network impairment start decades before the main cognitive symptoms appear (Jansen et al., 2015), they could be detected early in time by M/EEG. Their non-invasive nature allows the performance of as many recordings as needed in each subject, contrary to other neuroimaging modalities. This allows for detecting changes associated with healthy aging (Fernández et al., 2012; Porcaro et al., 2019), tracking the course of the disease and the assessment of pharmacological and non-pharmacological interventions.

## The Role of Electrophysiological Techniques in Detecting Alzheimer Disease

Until now, the use of M/EEG in dementia has had a minor role. In fact, its use in the elderly population has been limited to the discard of epileptogenic activity or the detection of generalized slow wave activity. In the last two decades great advances in the analysis of the information contained in the M/EEG temporal series have revealed fundamental pathological mechanisms that could constitute new signs for early diagnosis and prognosis in the AD process.

While the advanced stages of AD may be associated with functional disconnection (Stam et al., 2009), earlier stages may be apparent in terms of communication disruption such as hypersynchronization as revealed by MEG (Maestú et al., 2008; Bajo et al., 2010; Buldú et al., 2011). Indeed, MEG studies of patients with Mild Cognitive Impairment (MCI) found alterations in network organization across the cortex preceding clinical dementia (López et al., 2014; Maestú et al., 2015). Findings in resting state activity show a dual pattern of increasing and decreasing functional connectivity over prefrontal and posterior regions, respectively, mainly in the alpha band (Maestú et al., 2008; López-Sanz et al., 2017a). To assess the clinical value of MEG and explore in more detail whether hypersynchronization could be a hallmark of network disruption, an international consortium of MEG laboratories from five countries and three different continents was established (Magnetoencephalography International Consortium for the Study of AD; MAGIC-AD). This study evaluated MEG functional networks as a biomarker at the individual level in a blind study. A machine learning approach was used to classify MCI and healthy controls providing an accurate classification, over 80% (Maestú et al., 2015). Again, increased synchronization between anterior and posterior regions provided the better classification rate.

A common factor discovered in all these studies was an increased synchronization between prefrontal and posterior regions, reflecting an imbalance between excitation/inhibition, leading to spurious synchrony (see Table 1). In fact, higher synchronization did not provide better cognitive performance. Hyposynchronization in posterior regions (López-Sanz et al., 2017a) was also found, indicating a dual pattern, which could reflect two different mechanism: first hypersynchrony due to the loss of E/I balance (Busche and Konnerth, 2016) and then hyposynchrony reflecting degeneration (López-Sanz et al., 2017a).

**Table 1:** Studies of M/EEG in preclinical AD and MCI.

**References**	**Comparison**	**Sample size**	**Methodology**	**Result**
Babiloni et al., 2018	MCI vs. CN	75 MCI, 75 CN	Sources, Resting, EEG, FC	Widespread decreased alpha FC
Babiloni et al., 2016	AD vs. CN	19 AD, 40 CN	Sources, Resting, EEG, Activity	Higher delta and lower low frequency alpha activities. Association between hypometabolism and delta activity
Bajo et al., 2010	MCI vs. CN	22 MCI, 19 CN	Sensors, Task, MEG, FC	Increased long distance interhemispheric FC & decreased anteroposterior FC
Buldú et al., 2011	MCI vs. CN	19 MCI, 19 CN	Sensors, Task, MEG, Graphs	Increased network strength and outreach
Canuet et al., 2015	MCI	4 pMCI, 8 sMCI	Sources, Resting, MEG, FC	Dual increased/decreased FC pattern affecting limbic structures. Decreased FC associated with impaired axonal integrity
Cuesta et al., 2015	MCI vs. CN f(ApoE)	20 MCI0ε4, 16 MCI1ε4, 8 CN1ε4, 19 CN0ε4	Sources, Resting, MEG, FC	Decreased alpha and beta hippocampal and IPL FC in MCI. Decreased delta FC in ApoE34. Dual increased/decreased FC pattern affecting frontal/temporal regions
Dubois et al., 2018	SCD	88 SCDp, 230 SCDn	Sources, Resting, EEG, Power	Increase in alpha power over time in prefrontal areas in Aβ positive subjects at baseline
Fernández et al., 2002	AD vs. CN	15 AD, 19 CN	Sources, Resting, MEG, Power	Increased bilateral temporoparietal delta and theta power
Gonzalez-Escamilla et al., 2014	MCI vs. CN	29 MCI, 26 CN	Sources, Resting, EEG, FC	Decreased frontotemporal and parietal alpha FC
Hata et al., 2017	AD	14 AD	Sources, Resting, EEG, FC	Tau negative correlated with FC left frontal eye field and the right auditory
Jelic et al., 1997	MCI f(ApoE) vs. CN	17 MCI0ε4, 14 MCI1ε4, 10 MCI2ε4, 18 CN	Sensors, Resting, EEG, power, and FC	APOE ε4 seems to be associated with selective decreases in FC
Jelic et al., 1998	AD continuum	14 AD, 12 MCI, 14 CN	Sensors, Resting, EEG, Power	Association between tau and the a/d power ratio
Koenig et al., 2005	AD continuum f(GDS)	211 AD, 92 MCI, 70 SCD, 46 CN	Sensors, Resting, EEG, FC	Decreased FC in Alpha, Beta, and Gamma frequency bands, and increased delta FC
Kramberger et al., 2013	AD vs. MCI vs. SCD	131 AD, 285 MCI, 310 SCD	Sensors, Resting, EEG, Activity	Slower activity correlated with lower Aβ42/ptau ratio and higher total tau.
López-Sanz et al., 2016	MCI vs. SCD vs. CN	51 MCI, 41 SCD, 39 CN	Sources, Resting, MEG, Power	Decreased alpha power. MCI showed slowing in their alpha peak
López-Sanz et al., 2017a	MCI vs. SCD vs. CN	51 MCI, 41 SCD, 39 CN	Sources, Resting, MEG, FC	Increased alpha anterior FC and decreased posterior alpha FC
López-Sanz et al., 2017b	MCI vs. SCD vs. CN	69 MCI, 55 SCD, 63 CN	Sources, Resting, MEG, Graphs	SCD showed an intermediate degree (between MCI and CN) of network disruption in multiple parameters
Maestú et al., 2008	MCI vs. CN	15 MCI, 20 CN	Sources, Task, MEG, Activation	Bilateral higher activity in the ventral pathway
Maestú et al., 2015	MCI vs. CN	102 MCI, 82 CN	Sensors, Resting, MEG, FC	Enhanced fronto-parietal and interhemispheric broadband FC
Moretti et al., 2017	MCI	23 MCI	Sensors, Resting, EEG, Power	Subjects with increased Ha/La showed hypometabolism and higher ratio of conversion
Nakamura et al., 2017	CNp vs. CN	13 CNp, 32 CN	Sources, Resting, MEG, FC	Decreased local FC in Pcu. Increased FC between Pcu and both IPL
Nakamura et al., 2018	MCI vs. CNp vs. CN	28 MCI, 11 MCInoAD, 13 CNp, 17 CN	Sources, Resting, MEG, Power	Increased frontal alpha power in MCI and CNp. Increased frontal delta power in MCI vs. CNp. Global increased theta power in MCI
Smailovic et al., 2018	AD vs. MCI vs. SCD	197 AD, 230 MCI, 210 SCD	Sensors, Resting, EEG, power, and FC	Aβ42 correlated inversely with theta and delta power. Tau correlated negatively with alpha power. Alpha and beta FC correlated inversely with increased pathology
Stomrud et al., 2010	CN	33 CN	Sensors, Resting, EEG, Power	Association between theta power and tau CSF measurements
Teipel et al., 2018	SCD	63 SCDp, 255 SCDn	Sensors, Resting, EEG, FC	Non global significant results. Aβp participants showed enhanced alpha FC and diminished beta FC

*Unless otherwise expressly stated, the results summarized in the column “Result” are described referring to the first group in the corresponding column “comparison.” SCD, subjective memorry complain; CN, cognitive normal; MCI, mild cognitive impairment; Aβ, Amyloid beta; XX0,1,2ε4, XX participants with 0, 1, or 2 allele/s of Apoε4; FC, functional connectivity. MCInoAD, mild cognitive impairment not AD type; GDS, global dementia score; XXp, XX participants with positive amyloidosis; XXn, XX participants with negative amyloidosis; pMCI, progressive mild cognitive impairment; sMCI, stable mild cognitive impairment; Ha/La, high alpha/low alpha ratio; a/d, alpha/delta ratio; PCu, precuneus; IPL, inferior parietal lobe*.

An additional step was applying the principles of graph theory to MEG analysis to test whether this disruption in communication affected the architecture of the functional networks in early stages. López-Sanz et al. (2017b), found that the MCI patients showed a decreased small-worldness, clustering, and transitivity as well as increased modularity in theta and beta bands. Another group with Subjective Cognitive Decline (SCD) showed similar but smaller changes in clustering and transitivity, while exhibiting alterations in the alpha band in the opposite direction of that shown by MCI for modularity and transitivity. Clustering was disrupted as well in MCI and SCD. Additionally, it was observed that there is an increase in modular partition variability in both SCD and MCI in theta and beta bands. All these findings reflect a progressive loss of network architecture that is associated with worsening of cognitive symptoms.

One important issue that needed further research was the reliability of the results of the functional networks. This was necessary to be able to track different stages of AD continuum and to test the efficacy of pharmacological and non-pharmacological interventions. Thus, regarding the reproducibility of the functional network results, it was performed a study in which subjects underwent three MEG scans in three separate days and different functional connectivity metrics were assessed in the source space. The intraclass-correlation was very high for phase synchrony metrics, in different brain networks such as the motor and the somatosensory cortex, and the default mode network (Garcés et al., 2016a).

While MEG studies have found a dual pattern of hyper and hyposynchrony, a more consistent finding in EEG has been a reduced alpha to beta coherence (Jelic et al., 1997; Jeong, 2004; Fonseca et al., 2013). Gonzalez-Escamilla et al. (2014) showed a reduction of alpha interhemispheric coupling in MCI patients compared to controls, with this effect being greater in APOE4 carriers. Recently, Babiloni et al. (2018), using an advanced technique for functional connectivity analysis, showed intra and interhemispheric reduction of alpha band network functioning in AD patients that were able to distinguish them from healthy elderly subjects and Parkinson Disease patients. In early stages, such as MCI, patients again showed a decrease of alpha functional connectivity in several brain regions (Babiloni et al., 2018). Combining a small world metric and APOE4 positivity, Vecchio et al. (2018) showed a 91.7% accuracy for correct classification of a large cohort MCI patients who did or did not convert to AD. Finally, Dauwan et al. (2016) using an effective connectivity metric showed that the common posterior-to-anterior pattern of directed connectivity in controls is reduced in dementia with Lewy bodies patients in the alpha band, and in AD patients in the beta band.

In all these EEG studies, the most prominent profile was a decreased functional connectivity with consequences on network architecture. This discrepancy with MEG findings, i.e., the dual pattern of hypo and hypersynchrony, should be further investigated and evaluated as to why hypersynchrony is not being detected regularly in EEG studies. This could be due to differences in the population tested (more advanced cognitive impairment in EEG studies than in MEG) or because of technical issues (different source reconstruction methods, metrics of functional connectivity, or reference in EEG). Simultaneous M/EEG recordings in healthy elders and in SCD/MCI/AD subjects are needed to evaluate similarities and differences between both techniques in the same sample.

## M/EEG as a Biomarker for Conversion from MCI to Dementia

A crucial factor for considering M/EEG as a biomarker for AD is its capability to predict conversion across the different stages of the disease (preclinical-prodromal-dementia). There are very few longitudinal studies and all focused in the conversion from MCI to dementia (see Table 2). In two longitudinal studies (2 years of follow-up) with a similar sample, those MCI patients who developed dementia showed higher alpha band synchronization than those who remained stable (Bajo et al., 2012; López et al., 2014). The higher the synchronization between the anterior cingulate cortex and the posterior cortical regions, the higher the likelihood for developing dementia (López et al., 2014). Furthermore, patients that showed high levels of p-tau in the CSF and later on developed dementia showed again an increased synchronization between the medial temporal lobe and the anterior cingulate cortex in the beta frequency band (Canuet et al., 2015).

**Table 2:** Studies of M/EEG on progression along AD continuum.

**References**	**Comparison**	**Sample size**	**Methodology**	**Result**
Bajo et al., 2012	pMCI vs. sMCI	5 pMCI, 14 sMCI	Sensors, Task, MEG, FC	Increased parieto-occipital and frontal FC
Gouw et al., 2017	pSCD vs. sSCD; pMCI vs. sMCI	25 pSCD, 38 sSCD, 83 pMCI, 59 sMCI	Sensors, Resting, EEG, Power	pSCD showed higher delta and theta power and lower alpha power and peak frequency
Jelic et al., 2000	AD vs. pMCI vs. sMCI vs. CN	15 AD, 14 pMCI, 13 sMCI, 16 CN	Sensors, Resting, EEG, Power	MCI groups showed lower theta power than AD. pMCI showed higher theta and lower beta
Jovicich et al., 2018	MCI vs. MCInoAD	81 MCI, 63 MCInoAD	Sources, Resting and Task, EEG, Activity	Increased delta and theta and decreased low frequency alpha activities. Reduced parietal/posterior task activity
López et al., 2014	pMCI vs. sMCI	19 pMCI, 30 sMCI	Sources, Resting, MEG, FC	Increased alpha FC between right anterior cingulate and temporo-occipital regions
Luckhaus et al., 2008	pMCI vs. sMCI vs. AD	44 AD, 88 MCI	Sensors, Resting, EEG, Power	Decreased alpha power over posterior regions. 22% of MCI progressed to dementia
Moretti et al., 2011	pMCI vs. sMCI	18 pMCI, 14 DnoAD, 44 sMCI	Sensors, Resting, EEG, Power	Increased t/g ratio associated with dementia; increased a3/a2 ratio associated with AD
Poil et al., 2013	pMCI vs. sMCI	25 pMCI, 61 sMCI	Sensors, Resting, EEG, Power	Combination of EEG signatures in the beta band predicted conversion to AD
Rossini et al., 2006	pMCI vs. sMCI	24 pMCI, 45 sMCI	Sources, Resting, EEG, FC	Increased FC in several frequency bands
Vecchio et al., 2018	pMCI vs. sMCI	71 pMCI, 74 sMCI	Sources, Resting, EEG, Graphs	AD like small worldness pattern in pMCI

*Unless otherwise expressly stated, the results summarized in the column “Result” are described referring to the first group in the corresponding column “comparison.” SCD, subjective memorry complain; CN, cognitive normal; MCI, mild cognitive impairment; DnoAD, dementia non-due to AD; Aβ, Amyloid beta; XX0,1,2ε4, XX participants with 0, 1, or 2 allele/s of Apoε4; FC, functional connectivity; MCInoAD, mild cognitive impairment not AD type; GDS, global dementia score; XXp, XX participants with positive amyloidosis; XXn, XX participants with negative amyloidosis; pMCI, progressive mild cognitive impairment; sMCI, stable mild cognitive impairment; a3/a2, alpha3/alpha2 ratio; t/g, theta/gamma ratio; PCu, precuneus; IPL, inferior parietal lobe; pSCD, progressive subjective memory complain; sSCD, stable progressive memory complain*.

In EEG, Jelic et al. (2000) did not find differences between MCI converters and stable at the time they all were MCI, but a subsequent evaluation showed that converters increased their theta relative power and decreased beta in the temporal lobe. Subsequent studies with EEG were consistent in finding increased delta power over temporal regions in MCI converters compared with non-converters (Rossini et al., 2006). Alpha differences were found as well, with a reduced posterior power density (Luckhaus et al., 2008). However, a discrepant finding was found by Moretti et al. (2011) where converters showed an increased alpha3/alpha2 ratio. Finally, in healthy subjects who were CSF/amyloid (+) with subjective memory complaints, higher delta and theta power predicted conversion to cognitive impairment, however, no association was found with tau protein (Gouw et al., 2017). Regarding functional connectivity, Rossini et al. (2006) found increased fronto-parietal coherence in agreement with the increased functional connectivity values found in converters in MEG studies (López et al., 2014).

An interesting approach was the one proposed by Poil et al. (2013) in which they combine the information from multiple EEG biomarkers into a diagnostic classification index to improve the accuracy of predicting conversion from MCI to AD. By integrating six EEG biomarkers [Amplitude correlations with Cz in Beta (13–30 Hz); bandwidth of subject-specific Beta frequency; peak width of dominant beta peak; range of amplitude values in Beta (13–30 Hz); ratio between theta and alpha power; and alpha relative power normalized with 1–45 Hz broadband] into a diagnostic index the prediction achieved a sensitivity of 88% and specificity of 82%, compared with a sensitivity of 64%, and specificity of 62% of the best individual biomarker.

## The Relationship Between Findings on MEG and EEG in Dementia

The findings discussed thus far suggest an important potential for using the hyper/hyposynchronization phenomena detected with MEG as a non-invasive diagnostic biomarker. However, the use of MEG world-wide is still not as common as MRI or EEG, suggesting that it would be important to demonstrate how EEG can be used to detect the findings found on MEG. M/EEG data compatibility in epilepsy has already been demonstrated (Aydin et al., 2015; Hunold et al., 2016), but there are no papers indicating how functional network results in AD are compatible between MEG and EEG. This needs simultaneous recordings in healthy elderly subjects and in patients at different stages of the disease. Lacking these simultaneous recordings, we have to refer to the similarities between MEG and EEG on the previous findings reviewed above. Their correlation is fairly great in the majority of the studies, however, some discrepancies still exist, especially showing reduced functional connectivity in early stages of the disease in EEG (Babiloni, 2018), instead of a dual pattern dominated by hypersynchronization as showed in MEG studies (Jelic et al., 1997; Jeong, 2004; Koenig et al., 2005; Fonseca et al., 2013; López-Sanz et al., 2017a; Nakamura et al., 2017).

## M/EEG and Current Biomarkers of AD

It would be important for M/EEG to add additional information regarding the pathogenesis, diagnosis and prognosis of dementia, as well as to serve as a tool for tracking the course of the disease to evaluate the consequences of novel therapeutic interventions. The first step in this would be to show how their findings correlate with current biomarkers, such as cerebrospinal fluid (CSF) or PET measures of amyloid-beta.

There are still very few papers evaluating this compatibility. Recently, Nakamura et al. (2017) showed in a MEG study how deposits of amyloid protein alter network organizations in healthy elderly compared to amyloid negative elderly subjects. Functional connectivity was especially disrupted in regions with high amyloid deposits such as the inferior parietal lobe and the precuneus. In fact, there was a correlation between the high amyloid deposition and brain synchrony in those two regions. The importance of these results is that this network impairment preceded FDG-hypometabolism or brain atrophy. Therefore, hypersynchrony was detected in very early preclinical stages. In a subsequent analysis of the same data, they showed how local networks were also altered in association with amyloid depositions. Thus, alpha power was increased in prefrontal regions in correlation with high amyloid deposition in healthy elderly subjects. This time, brain magnetic activity was also recorded in MCI patients and showed increased alpha power over prefrontal regions where high amyloid deposition was detected (Nakamura et al., 2018).

In this review, we found just one paper attempting to correlate levels of p-Tau in the CSF with resting state MEG activity in patients with MCI. That study found a positive correlation between the levels of the p-tau protein and the functional connectivity values between medial temporal lobe and anterior cingulate cortex in subjects with prodromal AD (Canuet et al., 2015). This finding agrees with the tau neuropathology network-model, as described by Braak and Braak, and with recent ideas of “transneuronal neurodegeneration” (Fornito et al., 2015). The potential network alterations driven by transneuronal degeneration shape a unique framework to assess the cascade of neuropathological changes underlying the progression of AD using M/EEG-tau-PET. In this study, the authors showed a decrease in phase synchronization between the motor cortex and posterior cingulate cortex as well as between orbitofrontal region and temporo-occipital cortex. This decreased synchronization could indicate a functional disconnection in these MCI subjects. A reduction in posterior cingulate functional connectivity mediated by p-tau was also associated with impaired axonal integrity of the hippocampal cingulum as evaluated by diffusion tensor imaging, which could be understood as a functional diaschisis (Canuet et al., 2015). This supports the notion that the disruption of anatomical networks influences brain organization at the functional level (Kuczynski et al., 2010), perhaps contributing to the clinical manifestations of MCI (Pineda-Pardo et al., 2014).

Another important comparison necessary to better understand the role of MEG in dementia was to compare MEG results with genetic profiles of elderly subjects. One study investigated the modulation of functional networks in participants carrying the APOE4 allele, comparing healthy elders and MCI patients who did or did not carry the APOE4 allele. Healthy elderly carriers showed an increased synchronization between anterior and posterior regions while MCI carriers showed lower synchronization. This suggests that being a carrier of the ε4 allele increases the risk for developing dementia and as well increases the probability for showing hypersynchrony in key brain regions. MCI carriers showed higher network disruption with a decrease in functional connectivity in the same regions where healthy elderly carriers showed increased synchrony values (Cuesta et al., 2015).

All these studies indicate that hypersynchrony appears in preclinical stages (healthy elders with high amyloid deposition or carriers of APOE4 and in SCD subjects) and in later stages hyposynchrony is a sign of neurodegeneration. Thus, an early unbalance between excitation and inhibition mechanisms could responsible for excessive excitatory activity that may lead to neuronal impairment (Busche and Konnerth, 2016).

Regarding EEG and biomarkers, there are still few published papers. Comparing EEG and glucose metabolism by FDG-PET (Moretti et al., 2017), found hypometabolism in MCI patients with higher high-alpha/low-alpha power ratio. A compatible result was found by Babiloni et al. (2016) where higher activity of delta sources and lower activity of low-alpha sources was found in cortical regions with lower glucose metabolism. In another study, however, no significant relationship between glucose consumption and cortical alpha-phase coupling was found either in MCI ApoE4 carriers or non-carriers (Gonzalez-Escamilla et al., 2014).

Regarding amyloid-PET, two related studies did not find differences between amyloid-PET (+) and (–) individuals (Dubois et al., 2018; Teipel et al., 2018). Analyzing just the amyloid (+) subjects separately, they found an increased functional connectivity in regions with high amyloid deposition; however, the correlations were not statistically significant after correction for multiple comparisons (Teipel et al., 2018). Taking blood-amyloid samples, Gonzalez-Escamilla et al. (2014) found that elevated levels of Aβ1–42 correlated with decreased parieto-occipital functional connectivity in healthy subjects, but not in amnestic MCI subjects.

Jelic et al. (1998) were the first comparing CSF markers with EEG, finding a positive correlation between CSF tau levels and the alpha/delta power ratio in AD patients. Hata and coworkers (Hata et al., 2017) found a negative correlation between Aβ42 concentration and theta current source density in the right temporal region. Additionally, when they assessed total tau concentration, this parameter was negatively correlated with the lagged phase synchronization between the left frontal eye field and the right auditory area in the alpha band in patients with AD. Thus, the higher total tau the less functional connectivity in the alpha band. The increased local theta (associated with lower amyloid in CSF) and the reduced alpha network (associated with higher total tau), then, were indicators of functional impairment. These results were compatible with a previous EEG study where the combined ratio of the phosphorylated tau and Aβ42 showed a positive correlation with theta power in the right posterior electrodes (Stomrud et al., 2010; Kramberger et al., 2013). A recent EEG paper, showed increased resting state delta and theta rhythms and decreased low-frequency alpha (8–10.5 Hz) in MCI with positive biomarkers in the CSF (Jovicich et al., 2018). Similar findings were found by another independent study where lower Aβ42 levels and high p-tau were associated with high delta/theta and low alpha/beta power respectively, while global field synchronization was decreased in alpha and beta (Smailovic et al., 2018).

Considering all these EEG studies, there seem to be inconsistencies between amyloid-PET and CSF studies. However, the low number of studies published could increase variability of the results due to differences in methodologies employed in diagnosis and data analysis. EEG/CSF studies appear to provide a consistent result, i.e., an increased slow activity power in posterior regions correlates with pathology at different stages of the disease. These results are consistent with MEG studies (Fernández et al., 2002; Nakamura et al., 2018). However, EEG/MEG tends to also show reductions in alpha band power (Garcés et al., 2013; Babiloni et al., 2014; López-Sanz et al., 2016), and diminished complexity in the electrophysiological signals (Fernández et al., 2010; Bruña et al., 2012; Smits et al., 2016).

Functional connectivity findings with EEG and MEG in correlation with CSF are very compatible. A dual pattern of hyper- and hypoconnectivity associated with Aβ42 and tau were found in the two studies available. However, M/EEG and amyloid-PET studies are clearly incongruent as in MEG studies there are robust differences between amyloid (+) and (–) healthy elderly subjects (Nakamura et al., 2017, 2018).

## Discussion

M/EEG has shown differential profiles of functional connections between healthy elders and patients at different stages of the AD continuum. These profiles seem to correlate with cognitive scores and the clinical course of the disease, being able to predict which of the SCD and MCI patients will convert to dementia.

Very few studies have tested for correlations with current biomarkers. The few studies that have been done show a correlation of MEG with both amyloid-PET and CSF values and EEG with CSF. There is an association with the genetic risk factor most frequently associated with AD, i.e., being a carrier of an APOE4 allele. This correlation with current biomarkers was an important test for M/EEG to: (1) understand the basis of the hyper and hyposynchrony phenomenon and power reduction, and (2) to make possible its use in the clinical scenario. However, some discrepant findings between MEG and EEG indicate the need for a study where biomarkers would be acquired in the same sample of patients where simultaneous M/EEG recordings were being performed. A study testing whether hypersynchronization in the alpha band can also be consistently found in EEG in subjects with positive biomarkers at early stages is still needed. Hypersynchronization in the alpha band found in MEG at specific locations can also be tested and transferred to EEG in the context of a simultaneous recording, using the same metric for signal analysis.

Functional Magnetic Resonance Imaging (fMRI) can as well provide valuable information about the organization of brain networks (Bullmore and Bassett, 2011). In fact, multimodal studies with the same subjects have shown strong similarities between fMRI and MEG brain network topologies in alpha and beta frequency bands (Garcés et al., 2016b). fMRI has shown a disruption of brain network organization at different stages of the disease, essentially indicating a progressive disruption of the default mode network, both at the network level (Lim et al., 2014; Jones et al., 2015) and at the local level (Jovicich et al., 2018); being hub regions primarily impaired (Drzezga et al., 2011; Crossley et al., 2014). Furthermore, patients that convert from MCI to AD showed entorhinal/hippocampal hypoconnectivity in comparison to those that did not covert (Delli Pizzi et al., 2019). Interestingly, MCI patients who did not convert to AD or did not show CSF signs of disease showed hyperconnectivity between hippocampal/entorhinal with neocortical/subcortical regions (Delli Pizzi et al., 2019). These results are essentially in agreement with those found with electrophysiological techniques in certain frequency bands such as alpha and beta. Consequently, it is of interest to consider an integrative approach of the different potential biomarkers provided by M/EEG with those provided by fMRI. Poil et al. (2013), showed how the integration between six EEG biomarkers improve the classification between converters and non-converters. This approach could be as well be very beneficial for MEG biomarkers and for combining EEG and MEG findings to achieve a global neurophysiological index. By using deep learning approaches this combination of potential biomarkers could go beyond electrophysiological biomarkers including genetics, fMRI and MRI modalities as well as neuropsychological tests and life style factors. Compatibility is always subjected to non-redundant information. While M/EEG and fMRI data are compatible at certain frequency bands, there are others frequencies which seem to provide additional information. Conversely, fMRI provide information from deep brain regions where M/EEG sensors are nowadays not capable to achieve good signal to noise ratios. Therefore, these two views of brain functional activity are compatible as they provide complementary information (see Jovicich et al., 2018). As commented above, the use of deep learning approaches combining different sources of information could overcome the limitations of a single modality improving our knowledge of the structural and functional basis of AD.

Four main conclusions come from this review: (1) more studies are needed correlating M/EEG with current biomarkers to validate these neurophysiological techniques for a regular use in the clinical scenario; (2) multicenter, blind studies with biomarkers will provide reliability and accuracy values for classification; (3) include other sources of information such as genetics, PET, MRI, life style factors, and neuropsychological scores to achieve a more global view of the disease; and, (4) simultaneous M/EEG recordings in elderly subjects would test the compatibility of the functional connectivity results between these two techniques.

## Author Contributions

FM wrote the manuscript. All authors were designing the concept and structure of the paper and review the final manuscript.

### Conflict of Interest Statement

The authors declare that the research was conducted in the absence of any commercial or financial relationships that could be construed as a potential conflict of interest.
