# Corrigendum: The Importance of the Validation of M/EEG With Current Biomarkers in Alzheimer's Disease

**DOI:** 10.3389/fnhum.2019.00081

**Published:** 2019-03-06

**Authors:** Fernando Maestú, Pablo Cuesta, Omar Hasan, Alberto Fernandéz, Michael Funke, Paul E. Schulz

**Affiliations:** ^1^Laboratory of Cognitive and Computational Neuroscience, Center for Biomedical Technology, Universidad Complutense and Universidad Politécnica de Madrid, Madrid, Spain; ^2^Department of Experimental Psychology, Universidad Complutense de Madrid, Madrid, Spain; ^3^Magnetic Source Imaging Unit, Department of Pediatrics, McGovern Medical School, University of Texas Health Science Center, Houston, TX, United States; ^4^Electrical Engineering and Bioengineering Lab, Department of Industrial Engineering & IUNE Universidad de La Laguna, Tenerife, Spain; ^5^McGovern Medical School University of Texas Health Science Center, Houston, TX, United States; ^6^Department of Legal Medicine, Psychiatry, and Pathology, Universidad Complutense de Madrid, Madrid, Spain

**Keywords:** functional connectivity, mild cognitive impairment, EEG, MEG, healthy aging, Alzheimer's disease

In the published article, there was an error in affiliation for Paul Schulz. Instead of affiliation “4”, it should be affiliation “5”, the “McGovern Medical School University of Texas Health Science Center, Houston, TX, United States.”

The authors apologize for this error and state that this does not change the scientific conclusions of the article in any way. The original article has been updated.

